# An optimized method for high-titer lentivirus preparations without ultracentrifugation

**DOI:** 10.1038/srep13875

**Published:** 2015-09-08

**Authors:** Wei Jiang, Rui Hua, Mengping Wei, Chenhong Li, Zilong Qiu, Xiaofei Yang, Chen Zhang

**Affiliations:** 1 State Key Laboratory of Membrane Biology, School of Life Sciences; PKU-IDG/McGovern Institute for Brain Research, Peking University, Beijing 100871, China; 2 Key Laboratory of Cognitive Science, Laboratory of Membrane Ion Channels and Medicine, South-Central University for Nationalities, Wuhan 430074, China; 3 Laboratory of Molecular Basis of Neural Plasticity, Institute of Neuroscience, Chinese Academy of Sciences, Shanghai, 200031, China

## Abstract

Lentiviral technology has proven to be a powerful tool to express exogenous genes in dividing and non-dividing cells. Currently, most protocols for generating high-titer lentivirus require ultracentrifugation, which can be an instrumental barrier for routine operations in a laboratory. In this study, the effect of relative centrifugal force (RCF) on the concentration efficiency of the lentivirus was systematically explored, and it was found that sucrose gradient centrifugation with a relatively low speed (≤10,000 *g*) robustly produces a high-titer virus (up to 2 × 10^8^ TU/ml). The optimal sucrose concentration is 10%, and the recovery rate of the functional virus is greater than 80%. The infection efficiency of both concentrated and un-concentrated lentivirus decreases rapidly when the viruses are stored at 4 °C (τ ≈ 1.3 days) or subjected to multiple freeze-thaw cycles (τ = 1.1 rounds). In summary, we describe an efficient and easy-to-handle protocol for high-titer lentivirus purification.

The Human Immunodeficiency Virus (HIV)-derived lentivirus belongs to the Retroviridae family^1^. It is characterized by incorporating viral RNA into the DNA of dividing and non-dividing cells. To produce a lentivirus that is capable of infecting host cells, three types of vectors must be co-expressed in virus-producing cells (typically HEK293T cells): a backbone vector containing the transgene of interests and self-inactivating 3′-LTR regions, one construct expressing viral structure proteins, and one vector encoding vesicular stomatitis virus glycoprotein (VSVG) for encapsulation^2^. The latest generation of the lentivirus system further separates the Rev gene from other structural genes, offering increased biosafety by reducing the possibility of reverse recombination^3^. Nowadays, lentivirus technology has become one of the most efficient tools to deliver exogenous genes into various types of cells both *in vitro* and *in vivo*^4^,^5^,^6^,^7^.

The purification of high-titer lentivirus (>10^7^ TU/ml) from a large volume of virus-containing medium is crucial for the application of lentivirus. The routine concentration of the lentivirus requires ultracentrifugation with relative centrifugal force (RCF), typically exceeding 90,000 *g*^8^,^9^,^10^,^11^,^12^,^13^, to remove impurities and to ensure a high infection rate, especially for *in vivo* applications. This can be achieved only by an ultracentrifuge, which is not a common instrument in a lab. Moreover, the ability of low-speed centrifugation to produce a high-titer virus has not been thoroughly investigated. Thus, the relationship between RCF and the concentration efficiency of the lentivirus was systematically evaluated, and the present data showed that sucrose gradient centrifugation with a relatively low speed (≤10,000 *g*) produces a high-titer virus that is comparable to a commercially available virus purified with ultracentrifugation in both yield and titer. Moreover, the optimal sucrose concentration is 10%. Thus, an efficient protocol for high-titer lentivirus purification that is easily achieved with a regular lab centrifuge is described.

## Results

**Figure 1:**
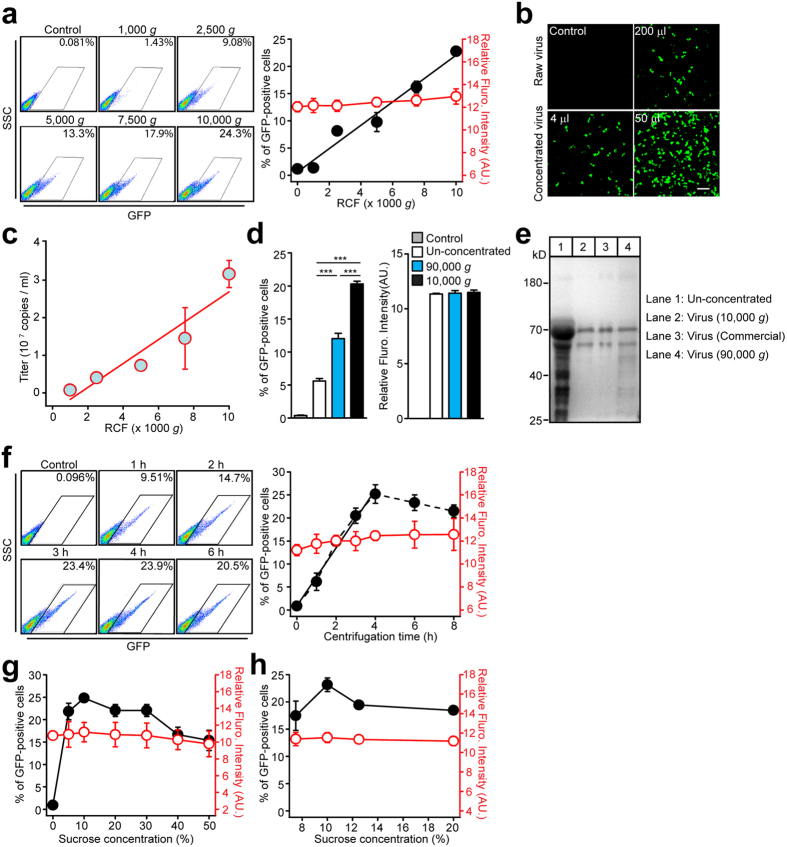
The examination of purification parameters on the concentration efficiency of the lentivirus. (**a**) Representative images (left) and summary graph (right) of the flow cytometric analysis of the HEK293T cells transduced with the GFP-expressing lentivirus purified with RCF. The HEK293T cells were trypsinized 36 hours after the transduction for the flow cytometric analysis. (**b**) Representative fluorescent images showing the HEK293T cells transduced with either a raw or concentrated GFP-expressing lentivirus. The viruses were enriched at a 1:50 ratio and added to the HEK293T cells at the volume indicated in the lower panels. Scale bar: 80 μm. (**c**) Summary graph of RT-PCR measurements of GFP-expressing lentivirus titer enriched with various RCFs at a 1:50 ratio. (**d**) Summary graphs of percentages and mean fluorescent intensities of the GFP-positive HEK293T cells transduced with either a raw or concentrated lentivirus enriched with 10,000 g centrifugation for 4 hours and 90,000 g for 1.5 hours, respectively. (**e**) Silver staining of equivalent amounts of the purified virus (1 × 10^5^ TU) prepared using either the RCF 10,000 *g* or ultracentrifugation (Commercial & RCF 90,000  g). (**f**) Representative images (left) and summary graph (right) of the flow cytometric analysis of the HEK293T cells transduced with the GFP-expressing lentivirus enriched with 10,000 *g* centrifugation for various durations. (**g**,**h**) Summary graph of the flow cytometric analysis of the HEK293T cells transduced with the GFP-expressing lentivirus enriched with 10,000 *g* with sucrose concentrations ranging from 0% to 50% (**g**) and 7.5% to 20.0% (**h**) in the centrifugation buffer. All summary graphs show mean ± SEM; n = 3 independent experiments (***p < 0.001).

To examine the lower limit of RCF, which could produce a reasonable yield of the high-titer lentivirus, the effect of RCF (1,000–10,000 *g*) on the concentration efficiency was examined. The HEK293T cells were transfected with a packaging vector expressing a green fluorescent protein (GFP) along with transfer and envelope vectors, and the virus-containing medium being collected at 48 hours after transfection was used as the starting materials for the concentration. To measure the titer of the functional virus, the concentrated virus was added to the HEK293T cells, and the percentage and relative intensity of the GFP-positive cells were measured with a flow cytometer (Fig. 1a). We used a 20% sucrose gradient centrifugation for 4 hours, which is most commonly used in published protocols, as the starting point. The concentration of functional virion particles, reflected as the percentage of the cells infected, was increased in a linear relationship with RCF in the range of 2,000–10,000 *g* (Fig. 1a,b). The virus purified with 1,000 *g* centrifugation showed almost no significant transduction ability in the HEK293T cells. The fluorescent intensity of the transduced HEK293T cells showed no significant differences in any of the concentrated viruses purified with various RCF tests. The concentration of viral genomes of the purified lentivirus was also measured using the RT-PCR method. Consistently, sucrose gradient centrifugation with a relatively low speed (≤10,000 g) robustly produces a high-titer virus in a RCF-dependent manner (Fig. 1c). The recovery yield of 10,000 *g* centrifugation was 85.6 ± 0.07% (suppl. Fig. 1). Intriguingly, 10,000 *g* centrifugation showed a 185.8 ± 23.7% transduction efficiency compared with the ultracentrifuge at the speed of 90,000 *g* for 90 minutes with otherwise identical settings (Fig. 1d). To test whether the different transduction efficiency was due to the different centrifugation time, the different speed, or both, a set of experiments were performed to compare the concentration efficiencies among the lentiviruses purified at various RCFs for various durations. The results show that the transduction efficiency had a linear relationship with the RCF in the range of 0–10000 *g*, and remained stable in the range of 10000–90000 *g* centrifugation for either 1.5 or 4 hours; however, the virus purified with centrifugation for 1.5 hrs always showed significantly less transduction efficiency compared with 4 hrs at all of the RCFs tested (suppl. Fig. 2). The impurity of the lentivirus preparations is an important issue, especially in *in vivo* applications. Thus, silver staining experiments were performed to examine the lentivirus purity. As shown in Fig. 1e, the virus purified using the low-speed centrifugation method contained as few contaminating proteins as the equivalent amounts of the lentivirus purchased commercially.

Because 10,000 *g* RCF could easily be achieved with most tabletop centrifuges and produces a superior yield of the concentrated virus, 10,000 *g* was set as the standard RCF for the optimization of other purification parameters. The effect of the centrifugation time on the virus transduction efficiency was first examined. As shown in Fig. 1f, the efficiency increased in a linear relationship with the centrifugation time and reached a plateau after 4 hours. Next, whether or not the sucrose concentration in the centrifugation buffer could affect the virus enrichment was tested. The osmotic of the centrifugation buffer was between 265.0 ± 3.00 mOsm/L and 2,143 ± 8.819 mOsm/L from 0% to 50% of the sucrose concentration in the buffer (suppl. Fig. 3). Without sucrose in the buffer, the transduction of the concentrated virus was very low. In the concentration window between 10% and 30% of sucrose, this purification method showed reliable performance in enriching the GFP-expressing lentivirus (Fig. 1g), and a more detailed analysis showed that 10% of sucrose exhibited the best effect in the virus concentration (Fig. 1h). Compared with other viruses, such as the Adeno-associated virus (AAV), the lentivirus is more fragile and subject to damage during purification. To test whether or not the acceleration or brake speed during centrifugation would affect the viability of the concentrated virus, two parameter sets—slow mode (acceleration and brake time of 146 and 172 seconds, respectively) and fast mode (acceleration and brake time of 62 and 35 seconds, respectively)—were tested when the centrifugation RCF was set to 10,000 *g*. The data showed that the virus concentrated in the slow mode was slightly but significantly more efficient (118.53 ± 6.81%) in infecting cells compared with the fast mode.

**Figure 2:**
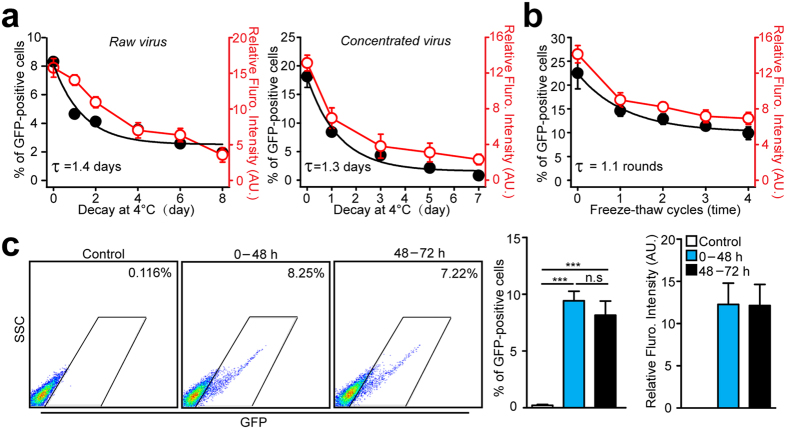
Optimization of lentivirus concentration/storage conditions. (**a**) Summary graphs of transduction efficiency as a function of storage duration at 4 °C. The percentages (black) and mean intensities (red) of the transduced cells were plotted to reflect the effectiveness of the raw (left) or concentrated (right) lentivirus. (**b**) Summary graphs of percentages (black) and mean intensities (red) of the HEK293T cells infected with the virus subject to multiple freeze-thaw cycles. (**c**) Representative images (left) and summary graph (right) of the flow cytometric analysis of the HEK293T cells transduced with the GFP-expressing lentivirus purified from virus-containing medium collected from the first 48 hours or 48–72 hours after transfection. All summary graphs show mean ± SEM; n = 3 independent experiments (***p < 0.001).

After concentration, the purified virus is typically either used for a short period of time or aliquoted and stored at −80 °C for long-term usage. Thus, the change in the virus titer upon prolonged storage of the lentivirus at 4 °C was tested. As shown in Fig. 2a, the infection efficiency of the un-concentrated and concentrated viruses was reduced exponentially (τ = 1.4 days and 1.3 days, respectively). The stability of the commercial-purchased virus was also measured, and it was found that the infection efficiency was reduced exponentially (τ = 1.9 days), which is similar to the un-concentrated virus and the concentrated virus purified by 10000 *g* purification (suppl. Fig. 4). Next, the effect of multiple freeze-thaw cycles on the infection efficiency of the virus was tested. During the test, the frozen virus was thawed at 4 °C for 20 minutes and added to the cells immediately at a 1:50 ratio. The results showed that the infection efficiency after one round of freeze and thaw was reduced by 33.3 ± 6.4% (τ = 1.1 rounds, Fig. 2b). To increase the yield of the lentivirus production, experimenters sometimes collect virus-containing medium from transfected HEK293T cells twice, once during the first 48 hours and again 48–72 hours after transfection. The results in this study consistently showed no significant difference in the infection efficiency or the fluorescent intensity of the cells infected with the viruses produced between 0 and 48 hours and between 48 and 72 hours after transfection (Fig. 2c).

**Figure 3:**
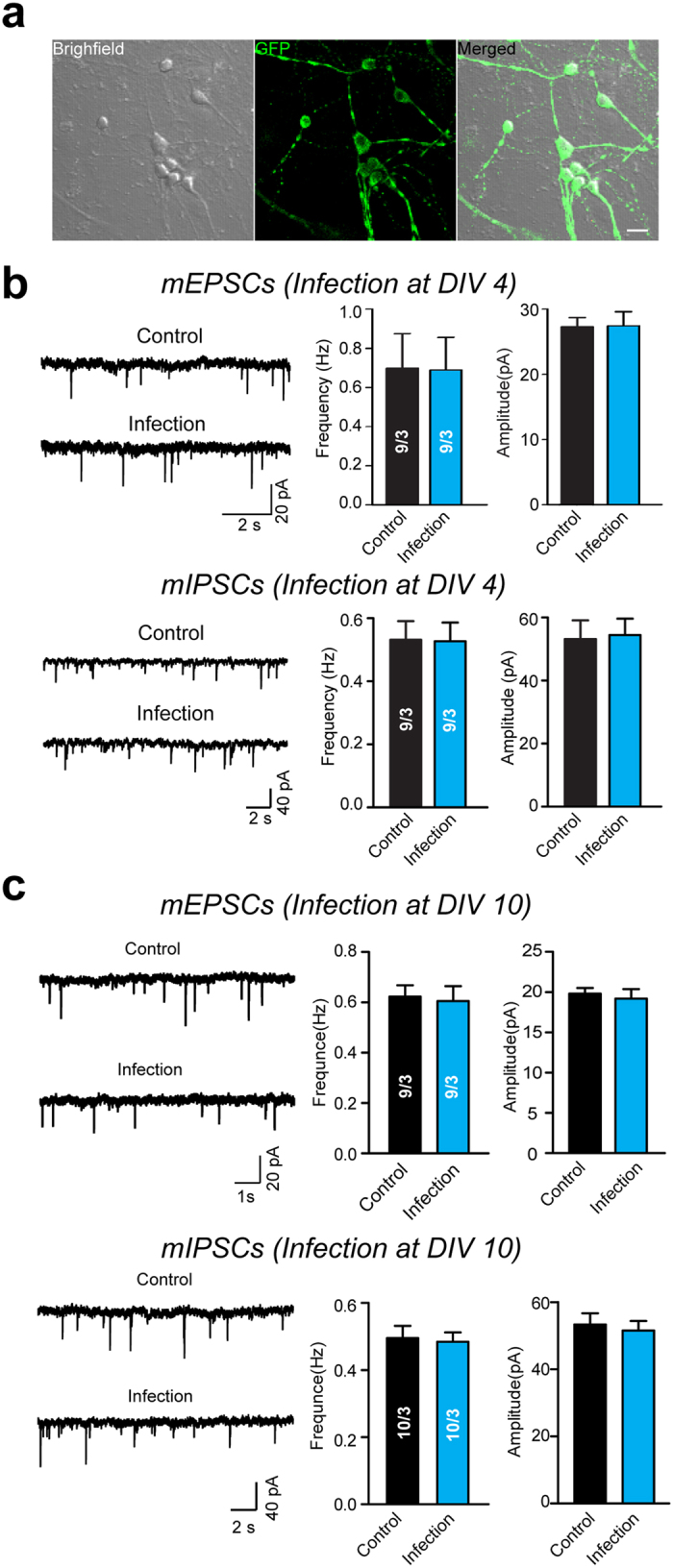
The lentivirus purified with 10,000 *g* centrifugation mediates the GFP expression in primary hippocampal neurons. (**a**) Representative brightfield (left), fluorescent (middle), and merged (right) images of cultured hippocampal neurons infected with the GFP-expressing lentivirus purified with 10,000 *g* centrifugation. Scale bar: 40 μm. (**b**) Representative traces (left), mean frequencies (middle), and amplitudes (right) of mEPSCs (top) and mIPSCs (bottom) recorded from neurons either infected with the concentrated GFP-expressing lentivirus (1:50 concentration, 4 μl) or treated with an equal amount of PBS at DIV 4. (**c**) Representative traces (left), mean frequencies (middle), and amplitudes (right) of mEPSCs (top) and mIPSCs (bottom) recorded from neurons infected at DIV 10. All summary graphs show mean ± SEM; n = 3 independent experiments.

**Figure 4:**
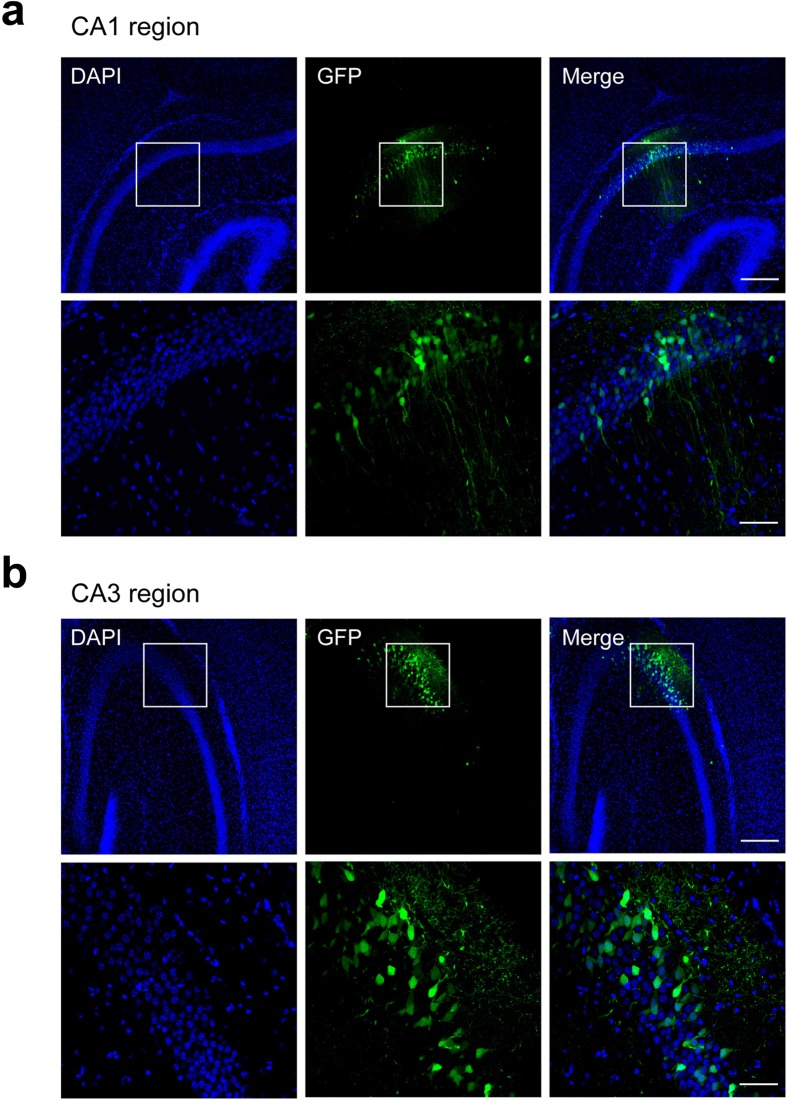
The lentivirus purified with 10,000 *g* centrifugation mediates the GFP expression in mice hippocampus. 1 μl virus (0.97 × 10^8^ TU/ml) was injected unilaterally into the hippocampus of new born mice pups (P0), and GFP expression was examined 12 days after injection in the cryostat sections. Low (upper panels in (**a**,**b**) Scale bar = 200 μm) and high (lower panels in (**a**,**b**) Scale bar = 50 μm) magnification views of brain sections show GFP-expressing neurons in CA1 and CA3 regions of hippocampus.

One of the major uses of the lentivirus is to infect non-dividing cells, such as neurons, for both *in vitro* and *in vivo* applications^14^,^15^,^16^,^17^,^18^. The virus purified with 10,000 *g* centrifugation was tested under both circumstances. The hippocampal neuronal cultures (8 × 10^4^ cells per coverslip in a 24-well plate) were infected with 4 μl of the virus (2.04 × 10^7^ TU/ml). As shown in Fig. 3a, almost 100% of the neurons showed GFP fluorescence at days *in vitro* (DIV) 14 when the concentrated virus was added at DIV 4. To test whether or not the concentrated virus may affect the growth of the neural cells, the miniature excitatory postsynaptic synaptic currents (mEPSCs) and miniature inhibitory postsynaptic synaptic currents (mIPSCs) of the infected neurons were measured and compared with the uninfected neurons. As shown in Fig. 3b, the mEPSCs and mIPSCs were normal both in frequencies and in amplitudes, implying that the addition of the concentrated virus did not cause detectable toxic effects to the neurons. In addition, the miniature synaptic transmission from the neurons infected with purified lentivirus at DIV 10 was measured when the synapses had already formed. The results show that the virus infection had no effect on the synaptic transmission, implying that the neurons in the late development stage (DIV 10) were not affected by the virus infection (Fig. 3c). Using a stereotaxic injection, the concentrated virus was injected into the CA1 and CA3 regions of the hippocampus in newborn mice pups. The GFP-positive CA1 and CA3 neurons were observed at P12, demonstrating the *in vivo* application of the method (Fig. 4). To further test whether or not the injected virus might result in a significant immune response, we detected the GFAP-positive astrocytes on hippocampal tissue slides from mice injected unilaterally with the lentivirus purified with 10,000 *g* centrifugations. The results show that no significant aggregation of GFAP-positive astrocytes were observed around the virus-injected sites in CA3 or CA1 region of hippocampus comparing to the uninjected hippocampus (suppl. Fig. 5). Thus, these results showed that the virus enriched with our method is capable of expressing exogenous genes in the non-dividing cells.

## Discussion

The purification of high-titer lentivirus is always a bottleneck for the application of lentivirus in a lab. Although other purification methods involving anion exchange HPLC^19^ or ultrafiltration^20^ have been reported, ultracentrifugation is still the most widely used method to concentrate lentivirus; however, the requirement of ultracentrifugation may represent an instrumental barrier in the concentration process. Here, the data demonstrated that the lentivirus can be concentrated with a sucrose gradient centrifugation at 10,000 *g* RCF, a centrifuge force easily achieved using a regular tabletop centrifuge. Furthermore, this method can be easily modified for the large-scale preparation of lentiviral stock. The titer of the concentrated virus in the results varied between 2.04 × 10^7^ TU/ml and 2.00 × 10^8^ TU/ml, largely depending on the volume of the initial materials. For example, with 50 ml of a virus-containing medium collected from transfected HEK293T cells, the concentration ratio is about 1:500, and the final volume is 100 μl. Using the high-titer lentivirus (1.32 × 10^9^ TU/ml), which is commercially purchased as the standard, the concentrated lentivirus prepared with a 1:500 concentration ratio reached a titer of 2 × 10^8^ TU/ml (suppl. Figs 6 and 7). Moreover, the data showed that the concentrated virus purified using this method does not affect the synaptic transmission in cultured primary neurons, which is a sensitive cell preparation (Fig. 3).

Several alternative methods have been reported to purify high-titer lentivirus preparations, which bypassed ultracentrifugation^19^,^20^,^21^,^22^,^23^,^24^,^25^,^26^,^27^. Ion exchange chromatography was used to purify lentiviral particles based on the fact that lentivirus are retained by the anionic exchange columns and can be eluted subsequently with buffers containing a high concentration of salts^19^,^25^,^26^. This method has the advantage of removing more cell debris compared with the ultracentrifugation. Ultrafiltration using Centricon units with a cut-off at 100 kDa in molecular weight was also reported to generate lentivirus vectors up to 10^9^ c.f.u./ml devoid of re-suspension problems in ultracentrifugation^20^. Polyethylene glycol (PEG) precipitation is another simple and efficient way to purify large volumes of lentivirus^21^,^27^. The method presented in this study is relatively simple and does not require special instrumentation or handling. With comparable transduction efficiency (∼10^8^ TU/ml), this method is relatively time-effective and especially suitable for handling a large variety of lentivirus purifications. In summary, a relatively simple and practical method to purify a high-titer and high-quality lentivirus for both *in vitro* and *in vivo* applications has been described.

## Methods

### Cell culture

The HEK293T cells (CRL-11268, ATCC) were grown at 37 °C and 5% CO_2_ in a humidified atmosphere incubator (Thermo). The culture medium contained Dulbecco’s modified Eagle’s medium (Gibco), 10% fetal bovine serum, and penicillin-streptomycin (50 μ/ml and 50 μg/ml). The HEK293T cells were seeded at the density of 100,000 per 8-mm × 8-mm, and the cover slip was coated with poly-lysine (Sigma) in a 24-well plate before transduction.

The dissociated hippocampal neurons were prepared from P0 pups, as described previously^14^. The neurons were plated at the density of 80,000 per 8-mm × 8-mm cover slip coated with poly-lysine (Sigma). The infected neuronal cultures were fixed with 4% paraformaldehyde/4% sucrose in phosphate buffered saline, and fluorescent images were collected with a laser confocal microscope (Olympus) using a 20× objective.

### Lentivirus package and concentration

Lentiviruses were produced by transfecting the HEK293T cells with the pFUGW vectors expressing GFP and three helper plasmids (pVSVg, RRE, and REV)^14^. The transfections were carried out using the Polyethylenimine (PEI) method with the ratio at PEI:pFUGW:pVSVg:RRE:REV = 24:3:1:2:2. The virus-containing medium was harvested 48 or 72 hours after transfection and subsequently pre-cleaned with a 3,000 *g* centrifuge and a 0.45 μm filtration (Millipore). The virus-containing medium was overlaid on a sucrose-containing buffer (50 mM Tris-HCl, pH 7.4,100 mM NaCl, 0.5 mM ethylene diamine tetra acetic acid [EDTA]) at a 4:1 v/v ratio and centrifuged at the indicated RCF at 4 °C. After centrifugation, the supernatant was carefully removed, and the tube was placed on the tissue paper for 3 minutes. Phosphate Buffered Saline (PBS) was added to the semi-dried tube for re-suspension, and then the tube was placed in the 4 °C fridge with a cover for recovery overnight.

### Flow cytometric analysis

The cells were washed once using PBS and digested with 0.05% Trypsin-EDTA (Gibco) for 1 minute at 37 °C. The dissociated cells were re-suspended in PBS for the flow cytometric analysis (BD). The data were analyzed using FlowJo 7.6 and Summit 5.0 software.

### Silver staining

Raw and concentrated virus proteins were extracted in the cell lysis buffer. The protein solution was denatured at 100 °C for 5 minutes and was separated on a 10% SDS–PAGE at 110 volts for about 1.5 hours. The gel was soaked in a 100 ml fixing solution for 120 minutes and was washed three times (5 minutes each) in distilled water. The gel was then put into a sensitizing solution with gentle shaking (55 rpm) for 60 minutes. Then, the gel was washed with distilled water three times. The silver solution was added to the gel and incubated with shaking for 60 minutes. After three washes with distilled water, the developing solution was added to the gel for six minutes, and the stop solution was added to terminate the reaction. Images of the gel were taken with a camera (SONY DSC-W630) after three more washes with distilled water.

### RT-PCR measurements of lentivirus titer

The enriched lentivirus was harvested, and the total RNA was isolated using kits (GeneCopoeia TM, Cat. No. HPR-LTK-050). In brief, 20.0 μl of lentivirus RNA was digested for 30–60 minutes at 37 °C to eliminate plasmid contamination. The total RNA (10.0 μl) was reversely transcribed using kits for 60 minutes at 37 °C. Real-time PCR amplifications were performed as following: (40 cycles) 95 °C for 10 minutes, 95 °C for 30 s, 60 °C for 1 minute, and 72 °C for 30 s. The copy number of the lentivirus was calculated based on the standard curve.

### Electrophysiological Recordings

Electrophysiological recordings were performed in whole-cell mode, and the synaptic currents were monitored with a Multiclamp 700A amplifier (Molecular Devices). Patch pipettes were pulled from borosilicate glass capillary tubes (World Precision Instruments) using a P-97 pipette puller (Sutter Instrument). The resistance of the pipettes filled with intracellular solution varied between 3 and 5 MΩ. The pipette solution contained 145 mM KCl, 5 mM NaCl, 10 mM HEPES, 5 mM EGTA, 0.3 mM Na_2_GTP, and 4 mM MgATP (pH 7.2, adjusted with KOH). The bath solution contained 150 mM NaCl, 4 mM KCl, 1 mM MgCl_2_, 2 mM CaCl_2_, 10 mM HEPES, and 10 mM glucose (pH 7.4, adjusted with NaOH). The miniature EPSCs were monitored in the presence of tetrodotoxin (TTX, 1 μM) and 100 μM picrotoxin. The miniature IPSCs were recorded in the presence of 1 μM TTX and 10 μM CNQX. The mEPSCs and mIPSCs were analyzed using Clampfit 10 software (Molecular Devices).

### *In vivo* injection of lentivirus and GFP expression detection

New born CD-1 pups (P0) were used in this study. All animal studies were conducted at the AAALAC-approved Animal Facility in the LAC-PKU. Experiments were undertaken in accordance with the guide for the care and use of laboratory animals (Eighth edition). All experimental protocols were approved by the Institutional Animal Care and Use Committee of Peking University. The injection procedure was described previously^28^. Briefly, pups were individually anesthetized on ice. Lentivirus was injected through glass micropipettes and stopped at the injection site for three minutes. At 12 days post-injection, mice were overdosed with sodium pentobarbitone (i.p.) and frozen brain sections were prepared as described previously^29^. GFAP staining was performed using GFAP antibody (1:2000, Neuromab 75–240).

### Statistical Analysis

The data are presented as mean and SEM. The statistical significance was determined by the Student’s t test.

## Additional Information

**How to cite this article**: Jiang, W. *et al.* An optimized method for high-titer lentivirus preparations without ultracentrifugation. *Sci. Rep.* **5**, 13875; doi: 10.1038/srep13875 (2015).

## Supplementary Material

Supplementary Information
