# Supplementary material for: An optimized method for high-titer lentivirus preparations without ultracentrifugation
Source: Sci Rep. 2015 Sep 8;5:13875. doi: 10.1038/srep13875 (PMC4562269; doi:10.1038/srep13875)
Supplement: Supplementary Information [file srep13875-s1.pdf]

Supplemental Information for Jiang et al., "An optimized method for high-titer lentivirus preparations without ultracentrifugation"

Wei Jiang [1,2,#], Rui Hua [1,2,#], Mengping Wei [1,2,#], Chenhong Li [2], Zilong Qiu[3], Xiaofei Yang [2]*, Chen Zhang[1]*

[1] State Key Laboratory of Membrane Biology, School of Life Sciences; PKU-IDG/McGovern Institute for Brain Research, Peking University, Beijing 100871, China

[2] Key Laboratory of Cognitive Science, Laboratory of Membrane Ion Channels and Medicine, South-Central University for Nationalities, Wuhan 430074, China

[3] Laboratory of Molecular Basis of Neural Plasticity, Institute of Neuroscience, Chinese Academy of Sciences, Shanghai, 200031, China

[#]These authors contributed equally to the work.

*Correspondence should be addressed to Y.X. (sunlittlefly@hotmail.com) and Z.C. (ch.zhang@pku.edu.cn).

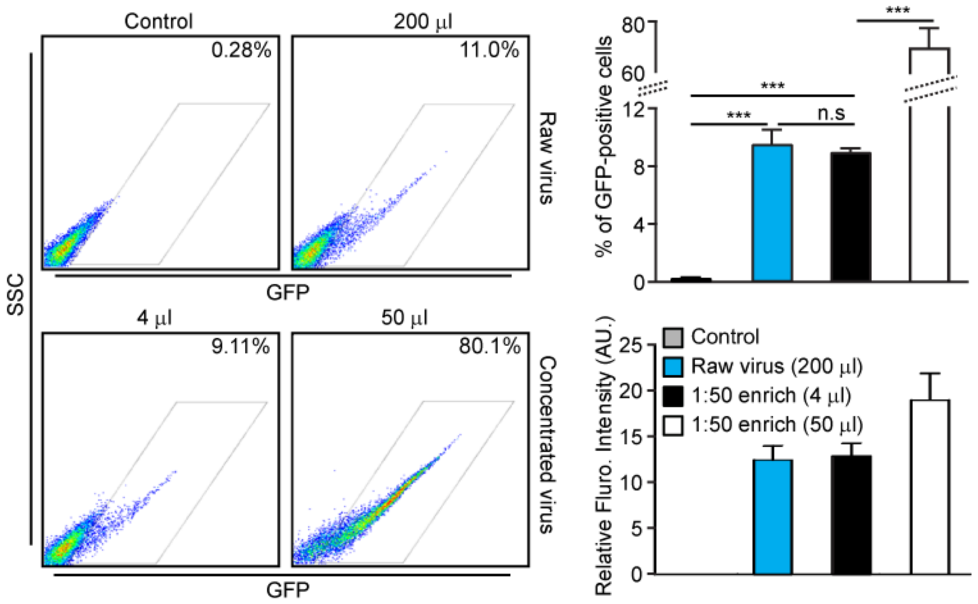

suppl. Figure 1. RCF on the concentration efficiency of the lentivirus. Representative images (left) and summary graph (right) of the flow cytometric analysis of the HEK cells transduced with either a raw or concentrated GFP-expressing lentivirus. The viruses were enriched at a 1:50 ratio and added to the HEK cells at the volume indicated in the panels. All summary graphs show mean ± SEM; n = 3 independent experiments (***, $p < 0.001$).

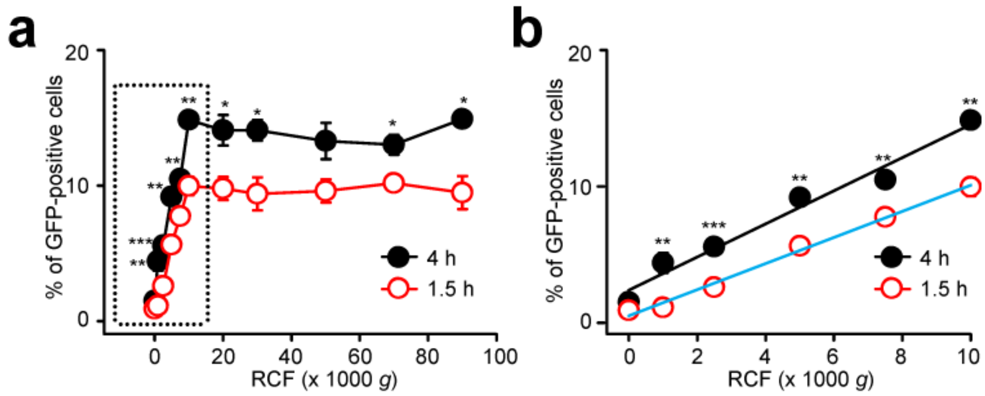

suppl. Figure 2. The plot of the transduction efficiencies of GFP-expressing lentivirus versus RCF and centrifugation time. **a** and **b.** Summary graph of the flow cytometric analysis of the HEK293T cells transduced with the GFP-expressing lentivirus purified with RCF for either 1.5 or 4 hours at a 1:50 ratio. All summary graphs show mean ± SEM; n = 3 independent experiments.

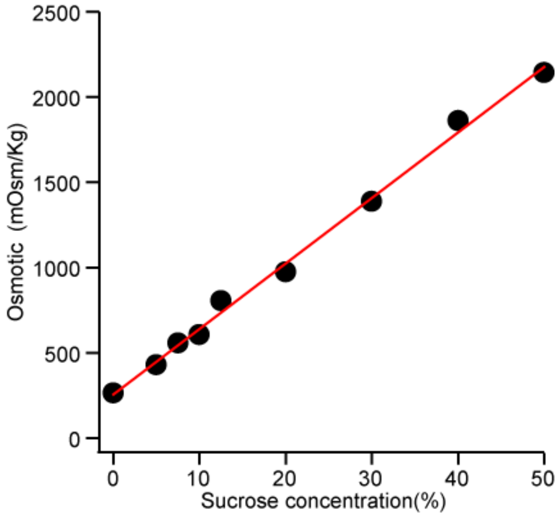

suppl. Figure 3. The osmotic of sucrose concentration in the centrifugation buffer. Summary graph of osmotic from 0% to 50% sucrose in the centrifugation buffer (50 mM Tris-HCl, pH 7.4, 100 mM NaCl, 0.5 mM EDTA). Summary graph shows mean ± SEM; n = 3 independent experiments.

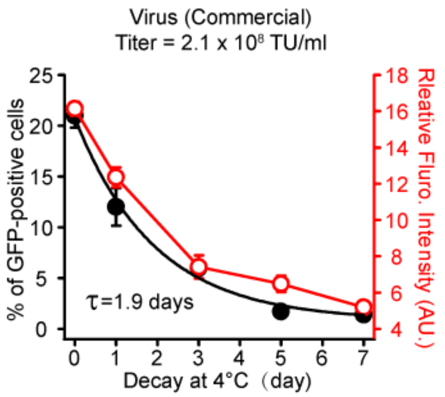

suppl. Figure 4. Measurements of the storage conditions of the transduction efficiency of commercial lentivirus as a function of storage duration at 4°C. Percentages (black) and mean intensities (red) of HEK293T cells were plotted to reflect the effectiveness of the commercial virus. All summary graphs show mean ± SEM; n = 3 independent experiments.

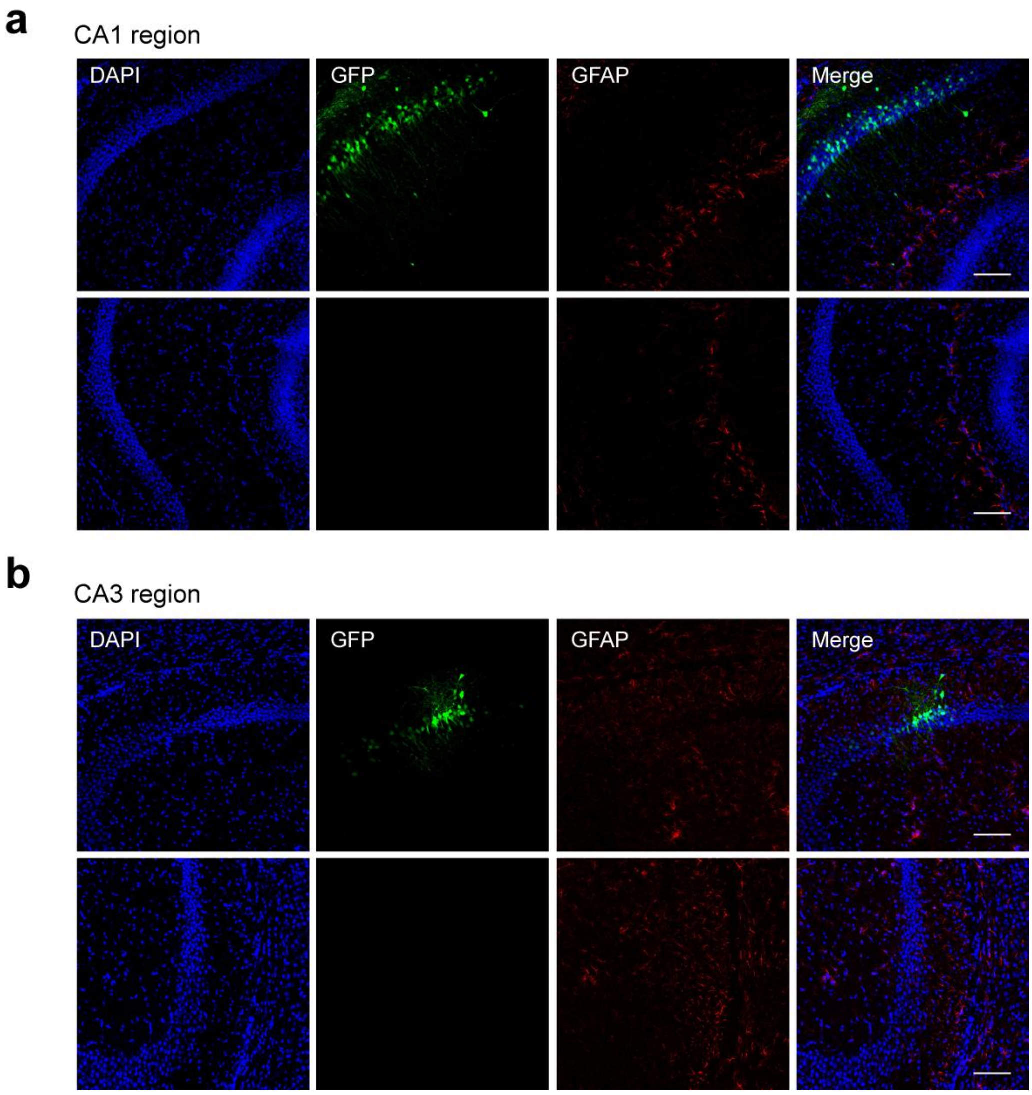

suppl. Figure 5. Detection of astrocytes with an antibody against GFAP on hippocampal tissue slides from mice injected unilaterally with the lentivirus purified with 10,000 *g* centrifugations. Scale bars: 100 μm. Images on the upper and lower rows in each panel represent the brain section from the injected hippocampus and contralateral uninjected hippocampus respectively.

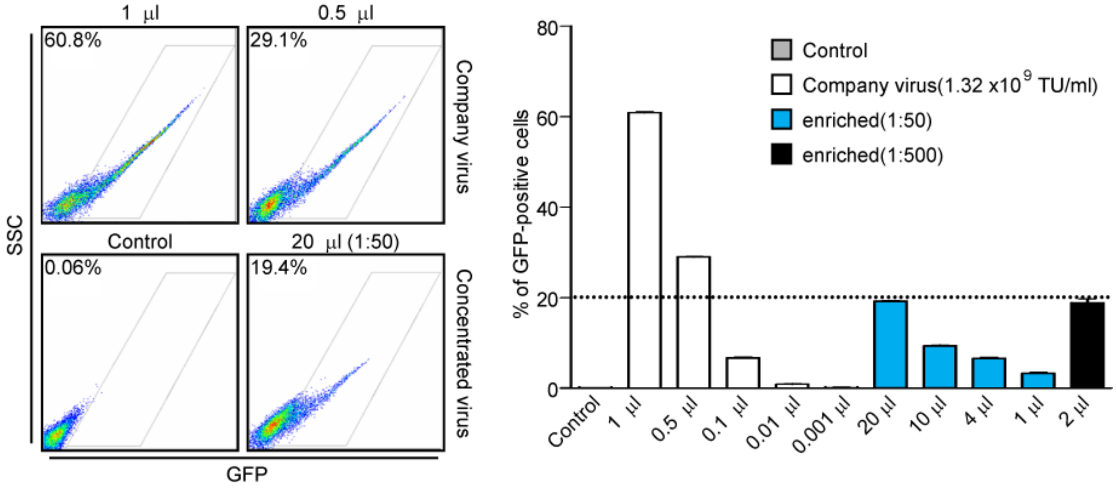

suppl. Figure 6. The titer of the concentrated virus compared with the commercially purchased virus as the standard. Representative images (left) and summary graph (right) of the flow cytometric analysis of the HEK cells transduced with the GFP-expressing lentivirus purified with sucrose gradient centrifugation with a relatively low speed or the commercially purchased lentivirus. The HEK cells were trypsinized 36 hours after the transduction for the flow cytometric analysis. All summary graphs show mean ± SEM; n = 3 independent experiments.

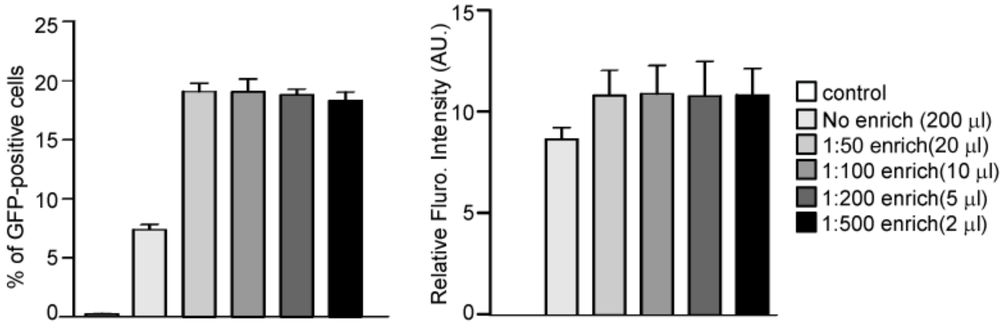

suppl. Figure 7. Sucrose gradient centrifugation with a relatively low speed (10,000 *g*) generates a high-titer lentivirus. Summary graphs of percentages (left) and mean fluorescent intensities (right) of the GFP-positive HEK cells transduced with the GFP-expressing lentivirus enriched with 10,000 *g* centrifugation at ratios ranging from 1:50 to 1:500. All summary graphs show mean ± SEM; n = 3 independent experiments.
